# Factors Affecting Outcomes in Anterior Cervical Spine Surgery

**DOI:** 10.1002/lary.70408

**Published:** 2026-02-06

**Authors:** Nana‐Hawwa Abdul‐Rahman, Hansen Deng, Jonathan Goulazian, Cynthia McMahan, Peter C. Gerszten, Carl H. Snyderman

**Affiliations:** ^1^ Department of Otolaryngology University of Pittsburgh School of Medicine Pittsburgh Pennsylvania USA; ^2^ Department of Neurosurgery University of Pittsburgh School of Medicine Pittsburgh Pennsylvania USA; ^3^ University of Pittsburgh School of Medicine Pittsburgh Pennsylvania USA

**Keywords:** ACDF, anterior cervical spine surgery, multidisciplinary approach, neck dissection, otolaryngology, surgical complication

## Abstract

**Objective:**

To evaluate procedure‐related complications after ACSS and identify key influential factors that increase or decrease the risk of perioperative complications.

**Methods:**

This single institution retrospective cohort study included 3401 patients who underwent ACSS by spine‐surgeons at a single institution from January 2015 to August 2023. Our main outcome of interest was perioperative complications after ACSS, divided into objective and subjective outcomes. Covariates included patient, procedure, surgeon, and institutional factors. Univariable and multivariable logistic regression were used to identify factors associated with complications.

**Results:**

Our cohort included 3401 patients averaging 55 ± 12 years and 50% (*n* = 1689) were male. The objective complication rate was 4.44% (*n* = 151) and subjective complications were 6.70% (*n* = 228). Otolaryngologists were involved in 17% (*n* = 591) of cases. On multivariable logistic regression, high volume (≥ 15 annual cases) surgeons (OR: 0.58, 95% Cl: 0.35–0.95, *p* = 0.030), and otolaryngologist involvement in revision surgeries (OR: 0.31, 95% Cl: 0.12–0.77, *p* = 0.011) were independent predictors of decreased odds of objective complications. Advanced age (OR: 1.04, 95% Cl: 1.02–1.05, *p* < 0.001) and surgeries involving multiple (≥ 3) spinal levels (OR: 1.70, 95% Cl: 1.23–2.34, *p* = 0.001) independently predicted an increased odd of subjective complications. Otolaryngologist involvement in upper (C1–4) cervical spinal cases (OR: 0.28, 95% Cl: 0.08–0.96, *p* = 0.042) independently decreased odds of subjective complications.

**Conclusion:**

Adopting a multidisciplinary approach with otolaryngologists as co‐surgeons in complex procedures and increasing surgeons' case volume may improve surgical outcomes, decrease complication rates, and improve quality of care.

**Level of Evidence:**

4

## Graphical abstract

This retrospective cohort study examined perioperative complications after anterior cervical spine surgery (ACSS) in 3401 patients treated at a tertiary academic institution. Otolaryngologist co‐surgeons in revision and upper cervical spine (C1‐4) cases, and high‐volume spine surgeons (≥ 15 annual cases) were associated with lower complication rates. Advanced age and multilevel procedures (≥ 3 levels) increased risk. Overall, these findings highlight the value of a multidisciplinary, team‐based surgical approach to improve outcomes in ACSS.

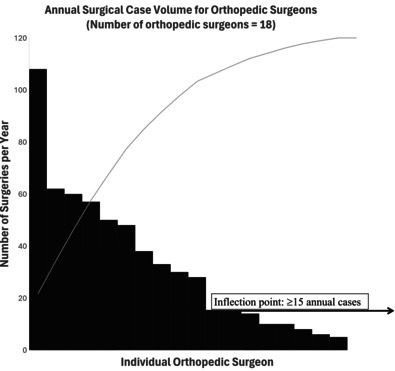

## 1 Introduction

Anterior cervical spinal surgery (ACSS) is widely used to treat various cervical spine pathologies, including neck trauma, degenerative disc disease, and neoplasia [1]. Initially popularized in the 1950s, ACSS has since become the gold standard for much cervical disc surgery [2, 3]. It provides better visualization and access to the intervertebral discs and has proven to be safe and effective [4, 5]. Despite these benefits, ACSS carries inherent risk due to proximity to other organs and critical neurovascular structures of the anterior cervical region. During surgery, critical neurovascular structures must be displayed to access the anterior cervical spine [6]. Unintentional injury to the carotid artery, jugular vein, vagus nerve and its laryngeal branches, esophagus, and trachea can result in long‐term severe morbidity and increase cost [7].

ACSS is traditionally performed alone by neurosurgeons and orthopedic surgeons. At some institutions, otolaryngologists may be involved in surgical site exposure for complex cases, such as revision surgery, morbidly obese patients, and patients with other neck pathologies [6, 8, 9, 10]. While previous studies have addressed postoperative subjective outcomes, specifically dysphagia and dysphonia, the impact of the otolaryngologist co‐surgeon on non‐subjective intraoperative and postoperative complications has not been thoroughly investigated [6, 8]. This study was performed in order to evaluate procedure‐related complications, defined as objective and subjective complications, after ACSS and identify key influential factors that increase or decrease complication rates.

## 2 Methods

### 2.1 Study Design and Population

This was a retrospective cohort study of 3401 patients who underwent ACSS by neurological surgery and orthopedic surgery from January 2015 to August 2023 at our institution. Primary and revision ACSS cases and complications were identified through the Clinical Data Warehouse using the International Classification of Disease, 10th revision (ICD‐10) codes (Table S1). A manual chart review of each patient's electronic medical record was conducted to refine the dataset and confirm: (1) surgical procedure was ACSS; (2) participation of otolaryngologist as co‐surgeon; (3) complications occurred postoperatively within a fixed time period (Table S1) after the ACSS procedure of interest but not prior to or unrelated to the ACSS procedure; (4) other complications not identified through ICD‐10 codes. Patients identified as having complications through ICD‐10 codes were re‐evaluated during chart review. Those without verified complications during chart review were classified as not having the complication. A revision surgery was considered as re‐operation after prior ACSS. This study was approved by the local Quality Improvement Committee (STUDY #3407).

### 2.2 Outcome Measures

The primary outcome was procedure‐related complications after ACSS. Complications were further grouped into subjective (dysphonia and dysphagia) within 90 days of ACSS and objective intraoperative: vertebral artery injury, carotid artery injury, esophageal injury, recurrent laryngeal nerve injury, or durotomy within 30 days of ACSS and objective postoperative: vocal cord paralysis, Horner syndrome, C‐5 palsy, surgical site infection, hematoma, new postoperative weakness, cerebrospinal fluid leak, wound dehiscence, or urinary retention within 90 days of ACSS.

### 2.3 Variables of Interest

Specific variables assessed as prognostic factors included patient demographics (age, sex, BMI), upper (C1–4) versus lower (C5–T1) spinal levels, number of spinal levels operated (≤ 2 vs. ≥ 3), diagnosis (degenerative disorders, neoplasm, trauma, and revision cases), otolaryngologist involvement in the procedure, primary surgeon specialty and case volume per year, and facility (academic vs. community hospital). Otolaryngologist involvement was defined as any case where otolaryngologists served as co‐surgeons by performing the surgical site exposure, after which the primary surgeon (neurosurgeon or orthopedic surgeon) completed the procedure. Otolaryngologist consultation for preoperative or postoperative management was not considered as otolaryngologist involvement.

### 2.4 Statistical Analysis

**FIGURE 1:**
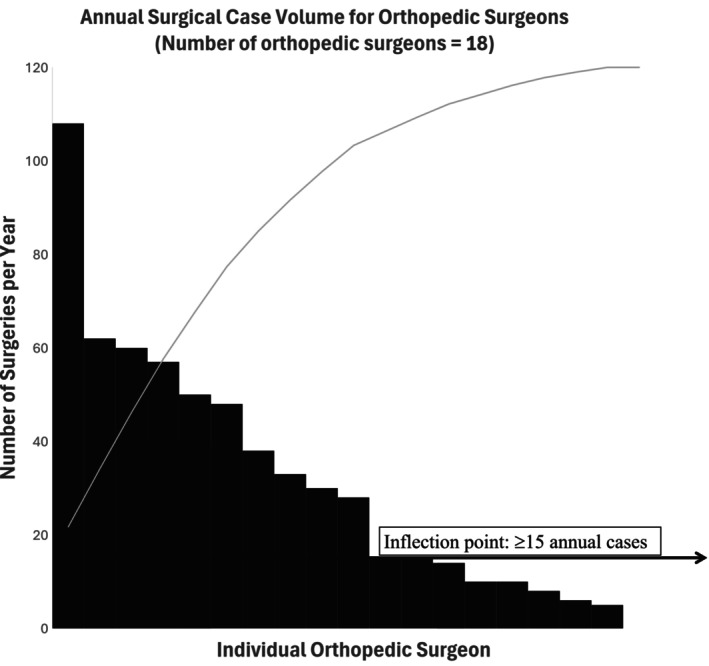
Distribution of annual orthopedic surgical volume per surgeon over the study time period.

**FIGURE 2:**
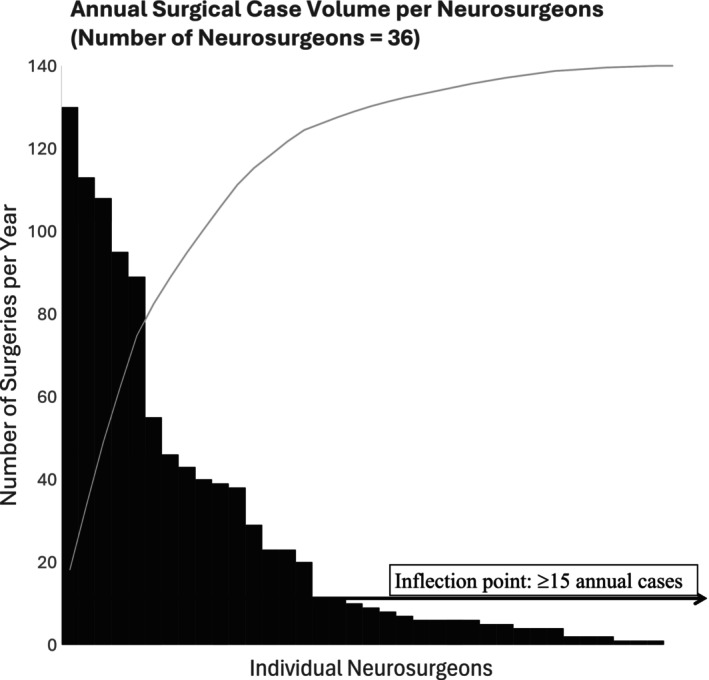
Distribution of annual neurosurgery surgical volume per surgeon over the study time period.

Statistical analyses were conducted using STATA, version 18 (Stata Corps, College Station, TX). Descriptive statistics were used to summarize the demographics of the patient population. Continuous variables are expressed as mean (SD) and categorical variables are expressed as frequency (%). An alpha of < 0.05 was considered statistically significant. Unadjusted logistic regression was used for univariable analyses. High‐volume surgeons were defined based on inflection points of volume histogram. For both neurosurgeons and orthopedic surgeons (Figures 1 and 2), inflection points were at 15 cases per year. Thus, surgeons that performed ≥ 15 cases per year were considered high‐volume surgeons. Multivariable logistic regression models were constructed to assess the effects of age, sex, BMI, disease type, primary surgeon's specialty, primary surgeon's volume, number of spinal levels, upper versus lower spinal level (upper = C1–C4 vs. lower = C5–T1), otolaryngologist involvement, and facility type on the odds of objective and subjective complications. Interaction terms were included to examine whether the effect of otolaryngologist involvement on the odds of complications varied by factors such as diagnosis and upper versus lower spinal levels. Multicollinearity was assessed, and the mean variance inflation factor was below 5. The features of both univariate and multivariable logistic regressions are presented as odds ratio (OR), 95% Cl, and *p*‐values.

## 3 Results

### 3.1 Demographics

In our cohort of 3401 patients, the mean age was 55 ± 12 years; 50% (*n* = 1689) of patients were male. As shown in Table 1, most of the cases were anterior cervical discectomy with fusion (ACDF) 99.4% (*n* = 3379). The majority were performed by neurosurgeons (67%, *n* = 2276), and 25% (*n* = 860) of cases were revision surgeries. The rate of subjective complications was 6.7% (*n* = 228) and objective complications were 4.4% (*n* = 151). Neurosurgeons performed more annual cases than orthopedic surgeons (Figures 1 and 2).

**TABLE 1:** Descriptive characteristics.

Patient characteristics	Mean (SD) or # (%, *N* = 3401)
Age	55.00 (12.49)
BMI	30.08 (6.51)
Annual case volume	
Neurosurgery	59.05 (32.38)
Orthopedic surgery	40.21 (15.27)
Biological sex	
Male	1689 (49.66)
Female	1712 (50.34)
Procedure	
ACC	22 (0.65)
ACDF	3379 (99.35)
Diagnosis	
Degenerative disorders	2402 (70.63)
Neoplasms	3 (0.09)
Revision	860 (25.29)
Trauma	136 (4.00)
Primary specialty	
Neurosurgery	2276 (66.96)
Orthopedic surgery	1123 (33.04)
+Otolaryngologist	591 (17.38)
Surgical facility	
Academic	2238 (65.80)
Community	1163 (34.20)
Cervical spinal levels	
Upper (C1–4)	256 (7.53)
Lower (C5–7)	3145 (92.47)
Number of spinal levels	
≤ 2	2685 (78.95)
≥ 3	716 (21.05)
Complications	
Subjective	228 (6.70)
Objective	151 (4.44)

Abbreviations: ACC: anterior cervical corpectomy; ACDF: anterior cervical discectomy and fusion.

### 3.2 Cases Involving Otolaryngologists

Otolaryngologists were involved in 17.4% (*n* = 591) of cases. Within the primary surgeon specialty, otolaryngologists were involved in 61.6% (*n* = 363) of neurosurgery cases and 38.4% (*n* = 226) of orthopedic surgery cases. Otolaryngologists were more likely to be involved in revision surgeries (63.6% vs. 17.2%, *p* < 0.001), surgeries of older patients (mean: 57 ± 12 years vs. 54.5 ± 12 years, *p* < 0.001), upper cervical spine cases (14.0% vs. 6.2%, *p* < 0.001), and spinal level ≤ 2 (84.4% vs. 77.8%, *p* < 0.001; Table 2).

**TABLE 2:** Subgroup analysis of procedures involving otolaryngologist.

Variable	Otolaryngologist involvement, yes *n =* 591	Otolaryngologist involvement, no *n =* 2810	*p*
Age, *mean (SD)*	57.21 (12.01)	54.54 (12.54)	< 0.001*
Disease type, *n* (%)			
Degenerative disorders	197 (33.33)	2205 (78.47)	< 0.001*
Neoplasms	3 (0.51)	0 (0)	
Revision Surgery	376 (63.62)	484 (17.22)	
Trauma	15 (2.54)	121 (4.31)	
Primary surgeon specialty, *n* (%)			
Neurosurgery	363 (61.63)	1913 (68.08)	0.002*
Orthopedic surgery	226 (38.37)	897 (31.92)	
Cervical spinal levels, *n (%)*			
Upper (C1–4)	83 (14.04)	173 (6.16)	< 0.001*
Lower (C5–7)	508 (85.96)	2637 (93.84)	
Spinal level ≤ 2, *n* (%)	499 (84.43)	2186 (77.79)	< 0.001*

*Note*: * Indicates significance: *p* < 0.05.

### 3.3 Factors Associated With Objective Complications: Unadjusted Logistic Regression

On univariate logistic regression (Table 3), factors associated with increased odds of objective complications include surgeries involving multiple (≥ 3) spinal levels (OR: 1.58, 95% Cl: 1.10–2.26, *p* = 0.013), revision cases (OR: 3.93, 95% Cl: 2.80–5.52, *p* < 0.001), and trauma cases (OR: 2.36, 95% Cl: 1.11–5.04, *p =* 0.026). Factors that decreased odds of objective complications include surgeries by high volume (≥ 15 annual cases) surgeons (OR: 0.55, 95% Cl: 0.34–0.88, *p =* 0.012), and otolaryngologist involvement in revision surgeries (OR: 0.31, 95% Cl: 0.12–0.76, *p =* 0.011).

**TABLE 3:** Univariate logistic regression of objective complications.

Variables	OR	95% Cl	*p*
Age^a^	1.02	1.00–1.03	0.013
Sex (Ref: male)			
Female	1.03	0.74–1.42	0.869
BMI^a^	1.00	0.97–1.02	0.908
Surgeon volume (Ref: < 15)			
≥ 15	0.55	0.34–0.88	0.012*
Surgical facility (Ref: academic)			
Community	0.71	0.49–1.02	0.063
Number of cervical spinal levels (Ref: ≤ 2)			
≥ 3	1.58	1.10–2.26	0.013*
Otolaryngology involved (Ref: no)			
Yes	1.35	0.91–2.01	0.139
Diagnosis (Ref: degenerative disorders and neoplasms)			
Revision surgery	3.93	2.80–5.52	< 0.001*
Trauma	2.36	1.11–5.04	0.026*
Cervical spinal level (Ref: lower [C5–7])			
Upper (C1–4)	1.17	0.65–2.09	0.606
Interaction: otolaryngology and diagnosis (Ref: degenerative disorders and neoplasms)			
Revision surgery	0.31	0.12–0.76	0.011*
Trauma	0.70	0.07–6.96	0.762
Interaction: otolaryngology and cervical spinal level (Ref: lower [C5–7])			
Upper (C1–4)	0.99	0.29–3.36	0.983

*Note*: * Indicates significance: *p* < 0.05.

Abbreviation: Ref: reference.

^a^ Continues variables.

### 3.4 Factors Associated With Objective Complications: Adjusted Logistic Regression

On multivariable logistic regression (Table 4), high volume (≥ 15 annual cases) surgeons (OR: 0.58, 95% Cl: 0.35–0.95, *p* = 0.030), and otolaryngologist involvement in revision surgeries (OR: 0.31, 95% Cl: 0.12–0.77, *p* = 0.011) remained independent predictors of decreased odds of objective complications.

**TABLE 4:** Univariate logistic regression of subjective complications.

Variables	OR	95% Cl	*p*
Age^a^	1.04	1.02–1.05	< 0.001*
Sex (Ref: male)			
Female	0.77	0.59–1.01	0.060
BMI^a^	1.00	0.98–1.02	0.717
Surgeon volume (Ref: < 15)			
≥ 15	0.81	0.52–1.26	0.348
Surgical facility (Ref: academic)			
Community	0.92	0.69–1.22	0.567
Number of cervical spinal levels (Ref: ≤ 2)			
≥ 3	1.51	1.12–2.04	0.007*
Otolaryngology involved (Ref: no)			
Yes	0.95	0.66–1.36	0.769
Diagnosis (Ref: degenerative disorders and neoplasms)			
Revision surgery	0.51	0.35–0.75	0.001*
Trauma	2.49	1.54–4.03	< 0.001*
Cervical spinal level (Ref: lower (C5–7))			
Upper (C1–4)	1.89	1.25–2.86	0.002*
Interaction: otolaryngology, diagnosis (Ref: degenerative disorders and neoplasms)			
Revision surgery	0.49	0.21–1.16	0.106
Trauma	0.80	0.19–3.36	0.759
Interaction: otolaryngology, cervical spinal level (Ref: lower [C5–7])			
Upper (C1–4)	0.38	0.13–1.13	0.081

*Note*: * Indicates significance: *p* < 0.05.

Abbreviation: Ref: Reference.

^a^ Continues variables.

### 3.5 Factors Associated With Subjective Complications: Unadjusted Logistic Regression

On univariate logistic regression (Table 5), factors associated with increased odds of subjective complications include advanced age (OR: 1.04, 95% Cl: 1.02–1.05, *p* < 0.001), surgeries involving multiple (≥ 3) spinal levels (OR: 1.51, 95% Cl: 1.12–2.04, *p* = 0.007), trauma cases (OR: 2.49, 95% Cl: 1.54–4.03, *p* < 0.001), and cases involving the upper (C1–4) cervical spinal levels (OR: 1.89, 95% Cl: 1.25–2.86, *p* = 0.002). Revision cases were associated with decreased odds of subjective complications (OR: 0.51, 95% Cl: 0.35–0.75, *p* = 0.001).

**TABLE 5:** Multivariable logistic regression of Objective complications.

Variable	OR	95% Cl	*p*
Age^a^	1.02	1.00–1.03	0.02*
Sex (Ref: male)			
Female	1.04	0.74–1.46	0.806
BMI^a^	1.00	0.98–1.03	0.932
Surgeon volume (Ref: < 15)			
≥ 15	0.58	0.35–0.95	0.030*
Surgical facility (Ref: academic)			
Community	0.74	0.50–1.09	0.128
Number of cervical spinal levels (Ref: ≤ 2)			
≥ 3	1.26	0.86–1.86	0.234
Interaction: otolaryngology, diagnosis (Ref: degenerative disorders and neoplasms)			
Revision surgery	0.31	0.12–0.77	0.011*
Trauma	0.58	0.05–6.23	0.653
Interaction: otolaryngology and cervical spinal level (Ref: lower [C5–7])			
Upper (C1–4)	1.31	0.37–4.60	0.678

*Note*: * Indicates significance: *p* < 0.05.

Abbreviation: Ref: reference.

^a^ Continues variables.

### 3.6 Factors Associated With Subjective Complications: Adjusted Logistic Regression

On multivariable logistic regression (Table 6), age (OR: 1.04, 95% Cl: 1.02–1.05, *p* < 0.001) and surgeries involving multiple (≥ 3) spinal levels (OR: 1.70, 95% Cl: 1.23–2.34, *p* = 0.001) remained independent predictors of increased odds of subjective complications. Otolaryngologist involvement in cases of the upper (C1–4) cervical spinal (OR: 0.28, 95% Cl: 0.08–0.96, *p* = 0.042) emerged as an independent predictor of decreased odds of subjective complications.

**TABLE 6:** Multivariable logistic regression of subjective complications.

Variable	OR	95% Cl	*p*
Age^a^	1.04	1.02–1.05	< 0.001*
Sex (Ref: male)			
Female	0.89	0.68–1.18	0.434
BMI^a^	1.01	0.99–1.03	0.259
Surgeon volume (Ref: < 15)			
≥ 15	0.85	0.53–1.35	0.482
Surgical facility (Ref: academic)			
Community	0.99	0.72–1.35	0.935
Number of cervical spinal levels (Ref: ≤ 2)			
≥ 3	1.70	1.23–2.34	0.001*
Interaction: otolaryngology and diagnosis (Ref: degenerative disorders and neoplasms)			
Revision surgery	0.59	0.25–1.42	0.238
Trauma	0.91	0.18–4.55	0.912
Interaction: otolaryngology, cervical spinal level (Ref: lower [C5–7])			
Upper (C1–4)	0.28	0.08–0.96	0.042*

*Note*: * Indicates significance: *p* < 0.05.

Abbreviation: Ref: reference.

^a^ Continues variables.

## 4 Discussion

### 4.1 Key Results

This study investigated rates of procedure‐related complications after ACSS and identified key influential factors. After adjusting for several factors including patient, procedure, and institutional level variables, we found that high volume surgeons and otolaryngologist involvement in revision cases significantly decreased the odds of objective complications. Otolaryngologist involvement in upper (C1–4) cervical spine cases decreased the odds of subjective complications while advanced age and surgeries involving ≥ 3 spinal levels increased the odds of subjective complications.

### 4.2 Interpretations

#### 4.2.1 Protective Factors, Objective Complications

This study demonstrated that a surgical team involving an otolaryngologist may decrease objective complications and help address the challenges of surgical site exposure in revision surgeries. Previous studies have only reported on the otolaryngologist role at decreasing subjective complications [6, 8]. Revision cases are more complex and often involve scarred tissue and altered neck anatomy—e.g., medialization of carotid artery. For this reason, meticulous surgical technique during site exposure is necessary to prevent objective complications that result from injury to critical structures of the anterior cervical region. Otolaryngologists' knowledge of operative neck anatomy and experience with dissection in all regions of the neck may facilitate tailored dissection techniques, with improved pattern recognition of anatomical relationships [11]. A recent study by Larkin et al. described a modified surgical technique to the neck dissection that was introduced by an otolaryngologist whereby instead of individually opening the three layers of the deep cervical fascia, the fascial layers were reflected medially through opening the carotid sheath and the neck dissection was performed to the spine in a lateral‐to‐medial fashion [11]. This approach is routinely used by otolaryngologist during oncologic neck dissection, laryngectomies, and cervical esophageal surgeries. According to the authors, this approach decreases tissue trauma and minimized the risk of inadvertent injury to critical neurovascular structures and thus can help decrease the rates of dysphagia [11]. However, because a larger dead space is created by the subplatysmal flaps, there is an increased risk of seroma formation [11]. We demonstrate that surgeries by high‐volume spine surgeons were associated with decreased objective complication rates. This is an intriguing, albeit unsurprising finding that mirrors previous investigations [12, 13]. Importantly, this protective effect was not seen in subjective complications. High‐volume surgeons are possibly more experienced, have greater familiarity with the procedure, and have refined operative techniques.

#### 4.2.2 Protective Factors, Subjective Complications

The results of this study demonstrate that otolaryngologist involvement in upper cervical spine procedures, defined as C1–4, is associated with a decreased likelihood of subjective complications, aligning with previous studies [6, 8]. This is an important finding, as it highlights an avenue to mitigate some of the most common complications after ACSS [1]. Dysphagia is a well‐known postoperative complications after ACSS, largely due to paravertebral edema, esophageal compression or retraction, and injury to the superior laryngeal nerve and recurrent laryngeal nerve [14]. Particularly, procedures involving the upper cervical spine levels (i.e., C1–4 levels) pose significant risk of injury to the superior laryngeal nerve whereas the recurrent laryngeal nerve is at greatest risk during dissection of the lower cervical spine levels [15]. Therefore, extensive knowledge of these structures with anatomic variation is essential at reducing postoperative dysphagia and dysphonia in ACSS [15].

#### 4.2.3 Predisposing Factors, Objective and Subjective Complications

Key factors associated with an increased likelihood of subjective complications included advanced age, involvement of upper cervical spine levels, and surgeries involving multiple spinal levels. On the other hand, revision surgeries, trauma surgeries, and surgeries involving multiple spinal levels increased the likelihood of objective complications. In a large scale study, Buerba et al. showed that advanced age independently increased complication rates even when controlling for patient comorbidities [16]. Older patients are more likely to have poor bone quality, multiple underling comorbidities, and sensitivity to anesthetic agents, factors that have been shown to increase surgical risk [17, 18, 19]. This highlights the need for factoring patient age in surgical decision making. Our findings of increased complications in surgeries of the upper cervical spine and multiple spinal levels is consistent with existing literature [20, 21]. Multiple spinal levels requires extensive dissection, tissue manipulation, and greater retraction (amount and duration) of soft tissues with possible compression of the endotracheal tube, which may exert greater pressure on the esophagus and larynx. Multiple spinal levels also involve the use of more hardware, which can later increase the potential for mechanical complications like hardware failure [22]. As previously discussed, dissection of the upper cervical spine increases the risk of neurovascular injury, contributing to both objective and subjective complications. Additionally, retraction of the larynx and trachea places significant tension on the structures, potentially leading to unrecognized neurovascular or mucosal injury.

### 4.3 Strengths of the Current Study

The diversity and volume of surgeries in our hospital system allows us to investigate prognostic factors at an institutional level. At our institution, otolaryngologists work in close proximity with neurosurgeons and orthopedic surgeons in many of our hospitals and often assist with complex cases. Our large sample size with shared electronic database and use of multivariable logistic regression models further strengthens the robustness of our findings. A detailed chart review of each patient's electronic medical record over the course of 1 year facilitated the generation of a precise and comprehensive dataset. The availability of data from multiple practice environments across our hospital network increases the generalizability of our findings. Importantly, we show that otolaryngologists improve outcomes for complex anterior cervical spine surgeries. While most of these procedures were performed in collaboration with high‐volume surgeons, our findings suggest that the benefit of otolaryngologist collaboration may be especially beneficial to low‐volume surgeons. Given their comparatively limited experience and reduced familiarity with the variable anatomy of the head and neck region, low‐volume surgeons are at high‐risk of complications, as evident by our findings and that of several other studies. Therefore, they may benefit the most from the protective effect of otolaryngologist collaboration.

### 4.4 Limitations

We recognize that despite having a large cohort in the current study, the retrospective nature can lead to inherent design bias. Since there were few factors that could be controlled in a prospective study, such as differences in surgical technique and anesthesia practices, we feel that a retrospective design was permissible. Lastly, we observed a low incidence of intraoperative complications which made it difficult to detect the impact of otolaryngologist involvement. The lack of routine assessment for laryngeal nerve injury and patient‐reported outcomes may underestimate the true impact of team surgery and surgical volume.

## 5 Conclusion

While ACSS carries significant risks, our study identified several modifiable factors that may improve outcomes. Our findings provide compelling evidence that increasing surgeon volume and leveraging otolaryngologist expertise for particular cases may effectively reduce the likelihood of complications, thereby improving patient outcomes.

## Funding

The authors have nothing to report.

## Disclosure

The authors have nothing to report.

## Conflicts of Interest

The authors declare no conflicts of interest.

## Supporting information


**Table S1:** Variables and their corresponding International Classification of Disease, 10th revision (ICD‐10) codes.

## Data Availability

The data that support the findings of this study are available on request from the corresponding author. The data are not publicly available due to privacy or ethical restrictions.
