# Supplementary material for: Factors Affecting Outcomes in Anterior Cervical Spine Surgery
Source: Laryngoscope. 2026 Feb 6;136(7):2963–70. doi: 10.1002/lary.70408 (PMC13253163; doi:10.1002/lary.70408)
Supplement: Supplementary file 1 — Table S1: Variables and their corresponding International Classification of Disease, 10th revision (ICD‐10) codes. [file LARY-136-2963-s001.docx]

**Supplementary Table 1: Variables and their corresponding International Classification of Disease, 10^th^ revision (ICD-10) codes**

| **Variable** | **ICD-10 codes** | **Timeline Considered**  **(from surgery to complications)** |
| --- | --- | --- |
| ACSS cases | 63075, 22554, 22551, 22552, 63075, 22554, 63075, 22845, 22846, L8699, 22851, 20930, 20931, 20936, 20937, 20938, 22856, 63050, 63051, 22558, 22585 | January 2015 – August 2023 |
| Vertebral artery injury | S15.1 | 30 days |
| Esophageal injury | S27.819A | 30 days |
| Neural injury (recurrent laryngeal nerve, superior laryngeal nerve) | J38.00 | 30 days |
| Horner's syndrome | G90.2 | 30 days |
| Durotomy | G97.41 | 30 days |
| Subjective dysphonia | R49.0 | 90 days |
| Dysphagia | R13.10 | 90 days |
| Hematoma | M79. 81, M79 | 90 days |
| C5 palsy | S14.115A | 90 days |
